# Cognitive Impairment following Mild Traumatic Brain Injury (mTBI): A Review

**DOI:** 10.3390/medicina60030380

**Published:** 2024-02-24

**Authors:** Ioannis Mavroudis, Alin Ciobica, Andreea Cristina Bejenariu, Romeo Petru Dobrin, Mihai Apostu, Irina Dobrin, Ioana-Miruna Balmus

**Affiliations:** 1 Department of Neurology, Leeds Teaching Hospitals, NHS Trust, Leeds LS2 9JT, UK; i.mavroudis@nhs.net; 2 Faculty of Medicine, Leeds University, Leeds LS2 9JT, UK; 3 Department of Biology, Faculty of Biology, Alexandru Ioan Cuza University of Iasi, Carol I Avenue 20th A, 700505 Iasi, Romania; alin.ciobica@uaic.ro; 4 Center of Biomedical Research, Romanian Academy, Iasi Branch, Teodor Codrescu 2, 700481 Iasi, Romania; 5 Academy of Romanian Scientists, 3 Ilfov, 050044 Bucharest, Romania; 6 Preclinical Department, Apollonia University, Păcurari Street 11, 700511 Iasi, Romania; 7 Department of Psychiatry, Faculty of Medicine, “Grigore T. Popa” University of Medicine and Pharmacy, 16th Universitatii Street, 700115 Iasi, Romania; andreea_costana@yahoo.com (A.C.B.); irina.dobrin@umfiasi.ro (I.D.); 8 Faculty of Pharmacy, “Grigore T. Popa” University of Medicine and Pharmacy of Iasi, 16th Universitatii Street, 700115 Iasi, Romania; mihai.apostu@umfiasi.ro; 9 Department of Exact Sciences and Natural Sciences, Institute of Interdisciplinary Research, Alexandru Ioan Cuza University of Iasi, Alexandru Lapusneanu 26th, 700057 Iasi, Romania; balmus.ioanamiruna@yahoo.com; 10 CENEMED Platform for Interdisciplinary Research, “Grigore T. Popa” University of Medicine and Pharmacy, 16th Universitatii Street, 700115 Iasi, Romania

**Keywords:** mild traumatic brain injury, cognitive impairment, neurocognitive assessment, neuroimaging, risk factors, cognitive rehabilitation, biomarkers

## Abstract

*Background:* Mild Traumatic Brain Injury (mTBI) has been increasingly recognized as a public health concern due to its prevalence and potential to induce long-term cognitive impairment. We aimed to consolidate this observation by focusing on findings of neuropsychological assessments, neuroimaging, risk factors, and potential strategies for intervention to prevent and treat mTBI-associated cognitive impairments. *Methods:* A thorough search of PubMed, PsycINFO, and Embase databases was performed for studies published until 2024. Studies focusing on cognitive impairment after mTBI, with neurocognitive assessment as a primary outcome, were included. *Results:* We found consistent evidence of cognitive deficits, such as memory and attention impairments, and affected executive functions following mTBI. Neuroimaging studies corroborate these findings, highlighting structural and functional changes in the brain. Several risk factors for developing cognitive impairment post-mTBI were identified, including age, gender, genetics, and pre-existing mental health conditions. The efficacy of interventions, including cognitive rehabilitation and pharmaceutical treatment, varied across studies. *Conclusions:* Mild TBI can lead to significant long-term cognitive impairments, impacting an individual’s quality of life. Further research is necessary to validate and standardize cognitive assessment tools post-mTBI, to elucidate the underlying neural mechanisms, and to optimize therapeutic interventions.

## 1. Introduction

Mild traumatic brain injury (mTBI) is a significant public health concern, affecting millions worldwide yearly. While most individuals fully recover within a few weeks, a substantial proportion of patients may experience persistent cognitive impairments that can negatively impact their quality of life [1].

The Mayo classification system defines mTBI as a type of brain injury that occurs when a person experiences a sudden blow or jolt to the head [2]. Patients receiving a Glasgow Coma Scale (GCS) score of 13 to 15 and experiencing loss of consciousness for less than 30 min and post-traumatic amnesia for less than 24 h are generally diagnosed with mTBI [2]. Mild TBI symptoms could last for days, weeks, or months and can significantly impact an individual’s daily functioning. Cognitive impairment following mTBI can manifest in various ways, including cognitive deficits, as well as increased emotional distress and somatic complaints [3]. Several studies have investigated the cognitive profile of individuals with mTBI during the acute phase and have reported significant impairment in global cognition, executive function, and episodic memory [4,5]. Diffuse axonal injury (DAI) is a major consequence of TBI, where the shearing forces during the injury event cause axonal damage. The severity of DAI correlates with the force of deceleration, and it can be identified within hours after the trauma. DAI is thought to play a significant role in cognitive impairment early after a mTBI [6,7].

Thus, we aimed to discuss the different cognitive domains that can be affected after mTBI, including executive function, attention, memory, and processing speed, in the acute phase and long-term follow-up after mTBI in various populations, including adults, children, and military personnel. These aspects could provide a comprehensive understanding regarding the nature and the extent of cognitive impairments after mTBI in order to improve the clinical management of patients, which includes the design of appropriate rehabilitation and support services. Also, it could provide a valuable resource for researchers, clinicians, and policymakers working in mTBI. Furthermore, we aimed to discuss the methods used to assess cognitive function after mTBI, including neuropsychological testing, neuroimaging, and electrophysiological measures, and to identify the risk factors for cognitive impairment after mTBI, which could further facilitate the development of targeted interventions to improve patient outcomes.

## 2. Materials and Methods

A comprehensive search of relevant studies on post-mTBI cognitive impairments that were published between 1990 and 2024 was performed in electronic databases, including PubMed, Embase, and PsycINFO, using a combination of keywords related to cognitive impairment, mild traumatic brain injury, and neuropsychological assessment. Inclusion criteria for the studies were (1) published in English, (2) conducted on human participants with mTBI, (3) included neuropsychological assessment of cognitive functions, and (4) reported results on cognitive impairment after mTBI. Exclusion criteria were (1) published in other languages, (2) not available in full text, and (3) not presenting relevant data on neuropsychological assessment of cognitive functions or cognitive impairment after mTBI.

## 3. Results

### 3.1. Timeframe and Duration of Cognitive Impairment in mTBI

The timeframe of cognitive impairment after mTBI can vary depending on the individual variability and the severity of the injury. Studies have shown that cognitive impairment and white matter damage can develop and persist over several years after a mTBI [8]. The evaluation of cognitive functions performed one month and 12 months post-injury have shown cognitive impairments in individuals receiving GCS scores of 13 to 15 [9]. Depression and anxiety are linked to poor cognitive performance in cases of complicated mild to severe TBI at 1-year post-injury [10] (Table 1). Thus, post-mTBI cognitive impairment can be prolonged and may persist for several years, highlighting the importance of monitoring and evaluating cognitive function in individuals who have experienced a mild TBI.

In addition to the severity of the injury, the duration of cognitive impairment after mTBI can also vary based on age, education, socioeconomic status, and previous history of TBI.

The most comprehensive tool to evaluate the post-TBI status in children is the SCAT (sports concussion assessment tool), which was previously designed for adults but adapted in various forms for infant patients [11]. This assessment tool comprises an extensive part for cognitive performance evaluation, including aspects of memory and attention capacity.

Studies have shown that children’s prognosis after mTBI is usually favorable, with quick symptom resolution and little evidence of residual cognitive deficits [9,10,12,13,14,15,16,17]. However, [9] few thoroughly discussed the implication of the appropriate control group when comparing cognitive impairments in children who have experienced TBIs. For instance, two of the studies [9] reported that hyperactivity and visual closure deficit could be associated with mTBI as a contributor factor rather than a consequence of the concussive event. Moreover, they discussed that a similar correlation may apply when assessing post-traumatic behavioral disturbances as a consequence of any injury rather than TBI, specifically. Caccese et al. [13] evaluated the neurocognitive performance in young football players and reported that repetitive concussions originating from childhood or adolescence do not predispose to cognitive impairment later in life due to developmental process disruption and cognitive reserve depletion.

Low overall cognitive function, records of previous TBIs, hospital admission for intoxications, and low education and socioeconomic status are strong risk factors for cognitive impairment after at least one mTBI [10,18]. In this way, it is important to monitor and evaluate cognitive function in individuals who have experienced a mTBI [19,20,21,22,23,24,25].

### 3.2. Impairment in Global Cognition

Many studies have reported global cognition impairment in patients with previous medical records of mTBI. Caccese and Iverson reported that 26.4% of patients with mTBI exhibited reduced global cognition as measured by the Mini-mental State Examination (MMSE) during the acute phase of the injury [26]. Similarly, McCrea et al. found that on the day of the injury, military service members with mTBI received a lower Military Acute Concussion Evaluation (MACE) cognitive score as compared to the non-concussed controls [5]. Altogether, these findings suggest that mTBI could predispose to global cognitive functioning deficits during the acute phase of the injury, which is generally defined as 0 to 3 days post-injury.

### 3.3. Executive Dysfunction and Episodic Memory Impairment

Executive dysfunction can also occur in the acute phase of mTBI, with deficits in attention, working memory, and cognitive flexibility. While evaluating active military service members diagnosed with mTBI, McCrea et al. found impairments in all cognitive domains on the day of the injury, including executive function [5]. It was reported that delayed memory deficits are typical in patients with mTBI, hinting at the complex cognitive processes that underlie these memory deficits [14]. Moreover, Wood et al. suggested that the executive dysfunction could be further linked to anomalies in social behavior, negatively impacting an individual’s capacity to live safely and independently in the community [15]. In the pediatric population, executive dysfunction could lead to significant issues across home, school, and community settings, with effects seen both in the short-term and long-term after injury [16].

In relation to executive memory functioning and other memory impairments, some recent studies have shown a possible significant correlation between cognitive impairment and the occurrence of headaches in post-concussion syndrome [17,18,19]. De Dhaem and Robbins reported that post-TBI headaches are often associated with poorer results in neurobehavioral evaluation, with regard especially to memory, attention, and processing speed [18]. However, long-term cognitive impairments were not associated with post-TBI headaches [18].

### 3.4. Attention and Working Memory

During the acute phase of mTBI, significant impairments in processing speed and attention have been reported [20]. It is worth mentioning that these cognitive deficits that occur in the first days post-injury may further lead to long-term functional impairment, threatening the quality of life [4].

However, one of the most frequently reported and clinically significant symptoms in mTBI patients is working memory deficits [21]. While the working memory involves the capacity to temporarily store and manipulate information that enables the accomplishment of a cognitive task, its impairment can substantially affect a person’s quality of life. Furthermore, the acute effects after mTBI may include post-traumatic amnesia, which may last up to 24 h. During the post-acute phase, memory deficits are usually the cause of increased distractibility, impaired attention, and deteriorated working memory [22]. Mild TBI-induced changes in working memory and functional activity have been reported even when differences in behavioral performance between mTBI patients and controls were absent, suggesting that cognitive assessment may increase sensitivity to evaluating post-mTBI symptoms, as compared to neuropsychological evaluation alone [23].

These findings underline the significance of both working memory and attention deficits following mTBI. Also, by being significantly detectable early after the injury, they offer important information for healthcare providers in their ongoing assessment and treatment of individuals with mTBI.

### 3.5. Subjective Cognitive Decline

Subjective cognitive decline refers to the self-reported perception of cognitive difficulties in daily life despite the absence of objective cognitive impairment on standardized neuropsychological testing. Research has attempted to investigate whether these self-reported cognitive symptoms after mTBI are associated with cognitive test performances, as well as whether the improvement in self-reported symptoms from 2 weeks to 3 months after mTBI is associated with an improvement in cognitive test performances [24,25]. A potential discrepancy between self-reported symptoms and objective test results has been noted, as patients with mTBI and persistent cognitive impairment for more than 3 months achieved normal scores in objective cognitive testing and vice versa [26]. Mild TBI patients displayed a significant decrease in cognitive performance and reduced stability of cognitive functions (as expressed by mean reaction time), as compared to controls, even when no symptoms of cognitive impairment were reported by the patients [26].

It has also been reported that comorbidities, and more specifically, anxiety and depression, may increase the severity and duration of self-reported symptoms in patients with normal objective tests [26].

### 3.6. Factors That May Influence Cognitive Impairment in mTBI

Several studies have investigated the relationship between the severity of mTBI and the risk of cognitive impairment. The previous reports suggested that cognitive impairment increases with injury severity, as moderate-severe TBI was associated with more severe cognitive dysfunction, while mTBI was associated with a higher risk of developing an anxiety disorder [27].

Older age has been identified as a risk factor for cognitive impairment after concussion as well as for long-term effects on cognitive performance [28]. A study found that older age was associated with worse cognitive outcomes after a mTBI, including slower processing speed, poorer working memory, and reduced attention [29]. Another study reported that older adults with a history of concussion had worse cognitive function than those without one, even after controlling for other factors, such as education and medical comorbidities [29]. Also, older adults with a history of concussion had a higher risk of mild cognitive impairment or developing dementia than those without a history of concussion [30,31].

It is important to note that while older age is a risk factor for cognitive impairment after concussion, not all older adults will experience cognitive deficits post-mTBI. Other factors, such as the severity of the concussion, the presence of other medical conditions, and individual differences in cognitive reserve, may also play a role in determining cognitive outcomes after a concussion. Thus, older age is a risk factor for cognitive impairment after concussion, and older adults may be more vulnerable to the long-term effects of concussion on cognitive function.

Depression and anxiety have been identified as risk factors for cognitive impairment after mTBI. Depression and anxiety were associated with cognitive impairment among individuals with complicated mild to severe TBI as long as after 1-year post-injury [32], while individuals with mTBI and comorbid depression or anxiety had worse cognitive outcomes than those without comorbid affective disorders [33]. Depression and anxiety may contribute to cognitive impairment after mTBI through several mechanisms. They can lead to changes in brain structure and function, including reduced gray matter volume and altered connectivity in brain networks involved in cognitive processing [31]. Additionally, depression and anxiety can interfere with attention, memory, and other cognitive processes, which may exacerbate cognitive deficits after mTBI [34].

Thomas et al. [35] reported that young athletes with co-occurring anxiety/depression had heterogeneous results of performance on memory tasks, suggesting cognitive decline. On the other hand, Delmonico et al. evaluated the potential risk for affective and behavioral disorders associated with mTBIs in the infant population and found that new psychiatric disorders may develop within 4 years post-injury in 10 to 13-year-old patients, posing consistent barriers to recovery from TBIs [35]. Moreover, Veliz and Berryhill recently suggested that there might be a sex-dependent difference in affective and behavioral disorder occurrence post-TBI [36]. In this way, they reported that girls are more prone to experience anxiety and attention deficits for new TBIs and aggression, social, thought, and conduct disorders following new and past TBIs, while boys experienced increased levels of anxiety and aggression, as a response to new and past TBIs [37].

### 3.7. Prognosis of Cognitive Impairment after mTBI

The prognosis of cognitive impairment following mTBI varies depending on the severity and frequency of the injury. Some studies have found that cognitive impairment can improve over time, mostly within the first year post-injury [30,38]. However, other studies have found that cognitive impairment can persist for many years following the brain injury, particularly in patients who have experienced repetitive concussions or have a history of previous head injury [31].

Besides the age, gender, education level, and comorbid medical conditions, the severity and persistence of cognitive impairment could also be influenced by the type of cognitive impairment, the deficits in executive function and working memory being particularly challenging to treat [39].

The prognosis for cognitive impairment following mTBI is variable and individualized, and treatment should be tailored to each patient’s specific needs and goals. Rehabilitation programs, cognitive training, and pharmacological interventions have all been shown to be effective in improving cognitive function in some patients, but further research is needed to determine the most effective treatments for this population.

### 3.8. Brain Areas Involved in Cognitive Decline after mTBI

Several studies have investigated the brain areas involved in cognitive decline after mTBI and reported that the disruptions in functional connectivity networks could predict cognitive impairment after acute mTBI [40]. While significant changes in rich-club organization and network properties were associated with early cognitive impairment after mTBI [40], cognitive sequelae were predicted by low-frequency connectivity alterations in large-scale brain connectivity [41]. The default mode network, which is involved in memory and attention, has been found to be disrupted after mTBI, potentially leading to cognitive deficits [41], especially attention deficits, which are a prominent component of cognitive dysfunction after mTBI [42]. White matter changes near cerebral microbleeds have also been associated with age and sex-dependent cognitive decline after mTBI [43].

D’Souza et al. investigated the association between cognitive dysfunction and alterations in resting-state functional connectivity in mTBI patients [43]. Thirty-three patients with mTBI and 33 healthy controls matched for age, gender, and education level underwent neuropsychological assessments and functional magnetic resonance imaging scans. The results showed that patients with mTBI exhibited significantly worse performance on measures of attention, executive function, and memory as compared to healthy controls. Furthermore, the mTBI group exhibited decreased resting state functional connectivity in several brain regions, including the left inferior frontal gyrus, left middle temporal gyrus, right inferior parietal lobule, and right middle occipital gyrus, significantly associated with cognitive dysfunction. The authors concluded that their findings provide evidence for the presence of alterations in resting state functional connectivity in mTBI patients, which may contribute to cognitive dysfunction, suggesting that these changes could be a potential biomarker for identifying and monitoring post-mTBI cognitive dysfunction.

Kinnunen et al. conducted a study to examine the long-term effects of mTBI on cognitive functioning and brain white matter integrity by evaluating 20 participants who had experienced mTBI at least one year before the study and 20 age- and gender-matched healthy controls. Both comprehensive neuropsychological evaluation and diffusion tensor imaging (DTI) were used to assess cognitive performances and brain white matter integrity, respectively [44]. They found that the mTBI group performed significantly worse than the healthy controls on measures of attention, working memory, and verbal learning and memory. In addition, DTI revealed significant differences in white matter integrity between the two groups: lower fractional anisotropy (FA) and higher mean diffusivity (MD) in several brain regions of mTBI patients, including the corpus callosum, internal capsule, and superior longitudinal fasciculus. In this context, these results could suggest that mTBI may lead to persistent cognitive deficits and changes in brain white matter integrity, even several years after the initial injury, highlighting the importance of long-term follow-up and monitoring of individuals who have experienced mTBI, as well as the need for further research to better understand the mechanisms underlying these cognitive and neural changes.

In this way, Niogi et al. conducted a study to examine the effects of mTBI on the structural integrity of white matter pathways in the brain. The authors used DTI to measure FA in the brains of 20 patients with mTBI and 20 healthy controls [45]. It was shown that mTBI patients were characterized by significantly lower FA in the corpus callosum and the cingulum, as compared to the control group. The corpus callosum is a central white matter tract that connects the left and right hemispheres of the brain, while the cingulum is a white matter pathway that is involved in attention, emotion, and memory. The study by Niogi et al. provides evidence that mTBI is associated with reduced white matter integrity in specific brain regions. The findings suggest that the corpus callosum and the cingulum are particularly vulnerable to injury in mTBI. These regions play essential roles in cognitive processes such as attention and memory, which may explain some of the cognitive deficits that are commonly observed in mTBI patients (Table 2). The results of this study have important implications for understanding the neural basis of mTBI and may help inform the development of interventions to improve outcomes for individuals with mTBI.

### 3.9. Biomarkers of Cognitive Impairment after mTBI

The research for biomarkers of cognitive impairment after mTBI has been driven by the need to develop objective and reliable methods for identifying patients at risk of developing post-mTBI cognitive impairments. Several biomarkers have been investigated in this context, including structural and functional imaging and biochemical markers in blood and cerebrospinal fluid.

Structural imaging techniques, such as magnetic resonance imaging (MRI) and DTI, have been used to investigate structural changes in the brain after mTBI. MRI has been used to detect lesions and changes in brain volume, while DTI has been used to investigate changes in white matter integrity. Several studies have reported that mTBI patients who develop cognitive impairment showed evidence of structural changes, including decreased volume in the hippocampus and thalamus and decreased white matter integrity in the corpus callosum and other white matter tracts [46,47]. Functional imaging techniques, such as positron emission tomography (PET) and functional MRI (fMRI), have also been used to investigate changes in brain function after mTBI. These techniques have been used to investigate changes in cerebral blood flow and metabolism, as well as changes in brain activity during cognitive tasks. Mild TBI patients who develop cognitive impairment show evidence of altered brain function, including decreased cerebral blood flow and metabolism in the frontal cortex and altered activity in the prefrontal cortex during cognitive tasks [48].

Biochemical markers in blood and cerebrospinal fluid have also been investigated as potential biomarkers of cognitive impairment after mTBI. These markers include various proteins and enzymes, such as tau, amyloid beta, and S100B. Several studies have reported that increased levels of these markers are associated with cognitive impairment after mTBI [49,50,51]. Newer studies have demonstrated that some point-of-care biomarkers could be used to determine the risk of developing prolonged recovery after a concussive event, which include cognitive impairments. However, Clarke et al. have shown that inflammatory biomarkers, such as IL-8, IL-9, IL-17a, TNFα, and monocyte chemoattractant protein 1, could be significantly correlated with the risk of developing persistent symptoms [52]. On the other hand, the results are quite heterogenous and controversial, as they have not singularly shown that some of the most relevant neuronal damage biomarkers—glial fibrillary acidic protein (GFAP) or neurofilament light (NFL)—levels changes might not be associated with prolonged symptoms or cognitive impairments due to concussive events [53,54,55]. For instance, a recent study on the effects of physical exertion on early changes in blood-based brain biomarkers reported that both GFAP and ubiquitin carboxyl-terminal hydrolase L1 (UCH-L1) levels might be influenced by physical activity and thus posing an important stint in TBI diagnosis in the first hours after the concussive event [53]. In this context, finding some reliable blood biomarkers for cognitive impairment in TBI still remains a matter of future perspective, as the changes in exosomal phosphorylated tau, NFL, IL-6, and TNFα might be more related to existent cognitive impairment rather than the history of TBI, as Ganesh and Galetti [56] commented in regards to the findings of Peltz et al. [57].

While biomarkers show promise as tools for diagnosing and prognosis of cognitive impairment after mTBI, more research is needed to establish their utility in clinical practice. Future studies should aim to identify reliable and sensitive biomarkers that can accurately diagnose and prognosticate cognitive impairment after mTBI.

### 3.10. Treatment and Management

Several approaches for treating and managing cognitive decline have been suggested in individuals with mTBI. These approaches include cognitive rehabilitation, medication, and lifestyle changes.

Cognitive rehabilitation is a common approach for treating cognitive impairment in individuals with mTBI. This approach involves structured activities designed to improve cognitive function, such as memory training, attention training, and problem-solving tasks. A meta-analysis found that cognitive rehabilitation effectively improves cognitive function in individuals with mTBI [58]. The 42 reviewed studies published between 2003 and 2008 gave significant evidence that cognitive rehabilitation interventions were associated with significant improvements in cognitive function, including attention, memory, and executive function, in individuals with mTBI.

Medication, such as methylphenidate and amantadine, can improve cognitive function in individuals with mTBI [59]. However, the use of medication for cognitive impairment in mTBI remains controversial, and more research is needed to determine the long-term effects of these medications.

Lifestyle changes, such as exercise and diet, have also been investigated as potential approaches for managing cognitive decline in individuals with mTBI. Several studies reported that routine physical exercises and a healthy diet can improve cognitive function in patients with post-mTBI cognitive impairment [60,61].

Overall, a multimodal approach that combines cognitive rehabilitation, medication, and lifestyle changes may be the most effective approach for treating and managing cognitive decline in individuals with mTBI (Table 3). However, more research is needed to determine the optimal combination of interventions and to identify which individuals may benefit most from each approach.

## 4. Discussion

**Figure 1:**
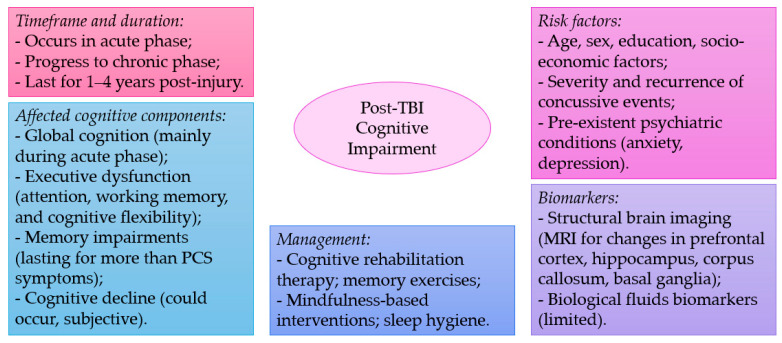
A summary of key points discussed in the current narrative review.

Mild TBI, also known as concussion, is a common type of brain injury that can occur due to various causes such as falls, sports injuries, work accidents, and motor vehicle accidents. While it is considered a mild form of TBI, it can still lead to various cognitive and behavioral symptoms. The studies discussed in this review highlight the prevalence of cognitive impairments following mTBI, with deficits reported in global cognition, executive function, attention, working memory, episodic memory, decision-making, and reaction times (Figure 1). Some studies have also identified brain areas associated with cognitive deficits in mTBI, including the prefrontal cortex, hippocampus, and corpus callosum.

Despite the prevalence of cognitive impairment in mTBI, effective treatments and management strategies for cognitive decline in mTBI remain limited. Studies have suggested that cognitive rehabilitation, exercise, and pharmacological interventions may help manage cognitive deficits in mTBI, but further research is needed to establish their efficacy. Moreover, studies have also suggested that mTBI may increase the risk of developing dementia in later life, highlighting the importance of early detection and management of cognitive impairments in mTBI.

The studies that were included in this analysis suggested that cognitive deficits can occur in various domains, such as attention, executive function, memory, and decision-making following mTBI. Furthermore, post-mTBI cognitive impairments can persist for several months or even years after the initial injury. The persistence of these cognitive deficits is alarming as they can significantly impact the quality of life of individuals, hindering their ability to perform daily activities and return to work.

The previous findings also suggested that the cognitive deficits associated with mTBI may be due to structural damage to specific brain regions, including the prefrontal cortex, hippocampus, and thalamus. These areas are crucial for cognitive processes, such as attention, working memory, and executive function. The damage to these regions may lead to the observed cognitive deficits in individuals with mTBI.

It is important to note that there is still much to be understood about the pathophysiology of cognitive deficits in mTBI. While some studies have identified specific brain regions, others have found more diffuse and widespread patterns of brain dysfunction. Moreover, individual differences in injury severity, age, and other comorbidities can significantly impact cognitive outcomes.

Management of cognitive impairment in individuals with mTBI should be multidisciplinary, involving healthcare providers such as neuropsychologists, occupational therapists, and rehabilitation specialists. The findings from the reviewed studies suggested that cognitive rehabilitation programs, including cognitive behavioral therapy and computer-based cognitive training, may be effective in improving cognitive outcomes in individuals with mTBI. However, further research is needed to determine this population’s most effective rehabilitation strategies.

The present review also highlights the potential risk for long-term sequelae, including the development of dementia. The reviewed studies suggest that there may be an increased risk for dementia in individuals with mTBI, particularly in those with a history of repeated head injuries. This highlights the need for long-term follow-up and monitoring of individuals with mTBI to identify and treat any cognitive impairment that may increase the risk of dementia.

However, there are some limitations worth mentioning. Firstly, future studies could evaluate more objectively the influence of mTBI on short and long-term cognitive performance in relation to neuroimagistics and biological analysis data. This could further characterize the correlation between mTBI and the mentioned cognitive impairments (memory, attention, affective, or executive functions, respectively). Also, the molecular changes observed in the brain could be the result of impairments in particular areas and could further better explain the origin of the noted cognitive impairment in relation to the molecular and signaling defects. Therefore, area-centered molecular analyses could shed more light regarding the pathomechanisms implicated in mTBI and subsequent cognitive impairments. Longitudinal studies on the risk of developing dementia or other neurodegenerative processes could also bring more evidence regarding the correlation between repetitive mTBI and neurodegeneration.

## 5. Conclusions

Cognitive impairments are a significant sequela of mTBI that can persist for an extended period of time and impact the quality of life of individuals. Previous studies suggested that cognitive deficits may be due to structural damage to specific brain regions, and cognitive rehabilitation programs may be effective in improving cognitive outcomes. Long-term monitoring and follow-up are needed to identify and treat any cognitive impairment that may increase the risk of dementia. Further research is needed to better understand the pathophysiology of cognitive deficits in mTBI and determine this population’s most effective rehabilitation strategies. Recommendations for future research include large-scale longitudinal studies, exploring the role of biomarkers in predicting cognitive outcomes, and developing personalized rehabilitation programs. Interdisciplinary collaborations can also enrich our understanding of the intricate dynamics between mTBI and cognitive impairment.

## Figures and Tables

**Table 1:** Types of cognitive impairment and timeframes in mTBI.

Cognitive Impairment Type	Timeframe
Global cognition	Acute
Executive function	Acute
Episodic memory	Acute
Attention and working memory	Acute to chronic
Decision making	Acute to chronic
Reaction times	Acute to chronic
Subjective cognitive decline	Chronic

**Table 2:** Brain areas involved in post-mTBI cognitive impairment.

Brain Area	Cognitive Impairment Type
Prefrontal cortex	Executive function, attention, working memory, decision making
Hippocampus	Episodic memory
Corpus callosum	Global cognition
Basal ganglia	Reaction times

**Table 3:** Management of cognitive decline in mTBI.

Management Strategy	Effectiveness
Cognitive rehabilitation therapy	Effective for executive function, attention, working memory, and episodic memory
Pharmacological intervention	Limited effectiveness
Exercise	Effective for global cognition, attention, and executive function
Mindfulness-based interventions	Effective for attention and working memory
Sleep hygiene	Effective for attention and working memory
Dietary interventions	Limited effectiveness

Note: Effectiveness may vary depending on the severity of cognitive impairment and individual differences. It is important to consult with a healthcare professional for personalized treatment and management.

## Data Availability

Not applicable.

## References

[1] Mavroudis I., Kazis D., Chowdhury R., Petridis F., Costa V., Balmus I.M., Ciobica A., Luca A.C., Radu I., Dobrin R.P. (2022). Post-Concussion Syndrome and Chronic Traumatic Encephalopathy: Narrative Review on the Neuropathology, Neuroimaging and Fluid Biomarkers. Diagnostics.

[2] Malec J.F., Brown A.W., Leibson C.L., Flaada J.T., Mandrekar J.N., Diehl N.N., Perkins P.K. (2007). The Mayo classification system for traumatic brain injury severity. J. Neurotrauma.

[3] McInnes K., Friesen C.L., MacKenzie D.E., Westwood D.A., Boe S.G. (2017). Mild Traumatic Brain Injury (mTBI) and chronic cognitive impairment: A scoping review. PLoS ONE.

[4] de Freitas Cardoso M.G., Faleiro R.M., de Paula J.J., Kummer A., Caramelli P., Teixeira A.L., de Souza L.C., Miranda A.S. (2019). Cognitive Impairment Following Acute Mild Traumatic Brain Injury. Front. Neurol..

[5] McCrea M., Guskiewicz K., Doncevic S., Helmick K., Kennedy J., Boyd C., Asmussen S., Ahn K.W., Wang Y., Hoelzle J. (2014). Day of injury cognitive performance on the Military Acute Concussion Evaluation (MACE) by U.S. military service members in OEF/OIF. Mil. Med..

[6] Spain A., Daumas S., Lifshitz J., Rhodes J., Andrews P.J., Horsburgh K., Fowler J.H. (2010). Mild fluid percussion injury in mice produces evolving selective axonal pathology and cognitive deficits relevant to human brain injury. J. Neurotrauma.

[7] Dikmen S., Machamer J., Temkin N. (2001). Mild head injury: Facts and artifacts. J. Clin. Exp. Neuropsychol..

[8] Miller D.R., Hayes J.P., Lafleche G., Salat D.H., Verfaellie M. (2017). White matter abnormalities are associated with overall cognitive status in blast-related mTBI. Brain Imaging Behav..

[9] Carroll L.J., Cassidy J.D., Peloso P.M., Borg J., von Holst H., Holm L., Paniak C., Pépin M. (2004). WHO Collaborating Centre Task Force on Mild Traumatic Brain Injury. Prognosis for mild traumatic brain injury: Results of the WHO Collaborating Centre Task Force on Mild Traumatic Brain Injury. J. Rehabil. Med..

[10] Nordström A., Edin B.B., Lindström S., Nordström P. (2013). Cognitive function and other risk factors for mild traumatic brain injury in young men: Nationwide cohort study. BMJ.

[11] (2017). Sport concussion assessment tool for childrens ages 5 to 12 years. Br. J. Sports Med..

[12] Laurer H.L., Bareyre F.M., Lee V.M., Trojanowski J.Q., Longhi L., Hoover R., Saatman K.E., Raghupathi R., Hoshino S., Grady M.S. (2001). Mild head injury increasing the brain’s vulnerability to a second concussive impact. J. Neurosurg..

[13] Caccese J.B., Houck Z., Kaminski T.W., Clugston J.R., Iverson G.L., Bryk K.N., Oldham J.R., Pasquina P.F., Broglio S.P., McAllister T.W. (2020). Estimated age of first exposure to American football and outcome from concussion. Neurology.

[14] Broadway J.M., Rieger R.E., Campbell R.A., Quinn D.K., Mayer A.R., Yeo R.A., Wilson J.K., Gill D., Fratzke V., Cavanagh J.F. (2019). Executive function predictors of delayed memory deficits after mild traumatic brain injury. Cortex.

[15] Wood R.L., Worthington A. (2017). Neurobehavioral Abnormalities Associated with Executive Dysfunction after Traumatic Brain Injury. Front. Behav. Neurosci..

[16] Kurowski B.G., Wade S.L., Kirkwood M.W., Brown T.M., Stancin T., Taylor H.G. (2013). Online problem-solving therapy for executive dysfunction after child traumatic brain injury. Pediatrics.

[17] Fan F., Anderson V., Morawakage T., Khan N., Shapiro J.S., Ignjatovic V., Takagi M. (2024). Post-traumatic headache pathophysiology in paediatric concussion: A systematic review. Neurosci. Biobehav. Rev..

[18] Begasse de Dhaem O., Robbins M.S. (2022). Cognitive Impairment in Primary and Secondary Headache Disorders. Curr. Pain. Headache Rep..

[19] Ashina H., Al-Khazali H.M., Iljazi A., Ashina S., Amin F.M., Lipton R.B., Schytz H.W. (2021). Psychiatric and cognitive comorbidities of persistent post-traumatic headache attributed to mild traumatic brain injury. J. Headache Pain..

[20] Arciniega H., Shires J., Furlong S., Kilgore-Gomez A., Cerreta A., Murray N.G., Berryhill M.E. (2021). Impaired visual working memory and reduced connectivity in undergraduates with a history of mild traumatic brain injury. Sci. Rep..

[21] Chung S., Wang X., Fieremans E., Rath J.F., Amorapanth P., Foo F.A., Morton C.J., Novikov D.S., Flanagan S.R., Lui Y.W. (2019). Altered Relationship between Working Memory and Brain Microstructure after Mild Traumatic Brain Injury. AJNR Am. J. Neuroradiol..

[22] Flynn F.G. (2010). Memory impairment after mild traumatic brain injury. Contin. Lifelong Learn. Neurol..

[23] Wylie G.R., Freeman K., Thomas A., Shpaner M., Okeefe M., Watts R., Naylor M.R. (2015). Cognitive Improvement after Mild Traumatic Brain Injury Measured with Functional Neuroimaging during the Acute Period. PLoS ONE.

[24] Stenberg J., Karr J.E., Terry D.P., Håberg A.K., Vik A., Skandsen T., Iverson G.L. (2020). Change in self-reported cognitive symptoms after mild traumatic brain injury is associated with changes in emotional and somatic symptoms and not changes in cognitive performance. Neuropsychology.

[25] Si T., Xing G., Han Y. (2020). Subjective Cognitive Decline and Related Cognitive Deficits. Front. Neurol..

[26] Rabinowitz A.R., Watanabe T.K. (2020). Pharmacotherapy for Treatment of Cognitive and Neuropsychiatric Symptoms after mTBI. J. Head. Trauma. Rehabil..

[27] Kubang K., Malaysia K., Sabarisah H., Nurshazwin R., Nasir M., Azrin M., Che Y. (2021). Association between pre-injury and injury-related factors and cognitive impairment of post-traumatic brain injury patients in a Hospital University Sains Malaysia cohort. IeJSME.

[28] Nelson L.D., Guskiewicz K.M., Barr W.B., Hammeke T.A., Randolph C., Ahn K.W., Wang Y., McCrea M.A. (2016). Age Differences in Recovery After Sport-Related Concussion: A Comparison of High School and Collegiate Athletes. J. Athl. Train..

[29] Covassin T., Stearne D., Elbin R. (2008). Concussion history and postconcussion neurocognitive performance and symptoms in collegiate athletes. J. Athl. Train..

[30] Gardner R.C., Burke J.F., Nettiksimmons J., Kaup A., Barnes D.E., Yaffe K. (2014). Dementia risk after traumatic brain injury vs. nonbrain trauma: The role of age and severity. JAMA Neurol..

[31] Crane P.K., Gibbons L.E., Dams-O’Connor K., Trittschuh E., Leverenz J.B., Keene C.D., Sonnen J., Montine T.J., Bennett D.A., Leurgans S. (2016). Association of Traumatic Brain Injury with Late-Life Neurodegenerative Conditions and Neuropathologic Findings. JAMA Neurol..

[32] Keatley E.S., Bombardier C.H., Watson E., Kumar R.G., Novack T., Monden K.R., Dams-O’Connor K. (2023). Cognitive Performance, Depression, and Anxiety 1 Year After Traumatic Brain Injury. J. Head. Trauma. Rehabil..

[33] Tanev K.S., Pentel K.Z., Kredlow M.A., Charney M.E. (2014). PTSD and TBI co-morbidity: Scope, clinical presentation and treatment options. Brain Inj..

[34] Kroes M.C., Rugg M.D., Whalley M.G., Brewin C.R. (2011). Structural brain abnormalities common to posttraumatic stress disorder and depression. J. Psychiatry Neurosci..

[35] Thomas G.A., Bradson M.L., Riegler K.E., Arnett P.A. (2023). Affective Disturbance and Neurocognitive Variability in College Athletes. Arch. Clin. Neuropsychol..

[36] Veliz P.T., Berryhill M.E. (2023). Gender Differences in Adolescents’ Affective Symptoms and Behavioral Disorders after Mild Traumatic Brain Injury. J. Head. Trauma. Rehabil..

[37] Delmonico R.L., Tucker L.Y., Theodore B.R., Camicia M., Filanosky C., Haarbauer-Krupa J. (2024). Mild Traumatic Brain Injuries and Risk for Affective and Behavioral Disorders. Pediatrics..

[38] Walker W.C., O’Neil M.E., Ou Z., Pogoda T.K., Belanger H.G., Scheibel R.S., Presson A.P., Miles S.R., Wilde E.A., Tate D.F. (2023). Can mild traumatic brain injury alter cognition chronically? A LIMBIC-CENC multicenter study. Neuropsychology.

[39] Li F., Lu L., Shang S., Hu L., Chen H., Wang P., Zhang H., Chen Y.C., Yin X. (2020). Disrupted functional network connectivity predicts cognitive impairment after acute mild traumatic brain injury. CNS Neurosci. Ther..

[40] Dunkley B.T., Da Costa L., Bethune A., Jetly R., Pang E.W., Taylor M.J., Doesburg S.M. (2015). Low-frequency connectivity is associated with mild traumatic brain injury. Neuroimage Clin..

[41] Alhourani A., Wozny T.A., Krishnaswamy D., Pathak S., Walls S.A., Ghuman A.S., Krieger D.N., Okonkwo D.O., Richardson R.M., Niranjan A. (2016). Magnetoencephalography-based identification of functional connectivity network disruption following mild traumatic brain injury. J. Neurophysiol..

[42] Irimia A., Ngo V., Chaudhari N., Zhang F., Joshi S., O’Donnell L., Sheikh-Bahaei N., Chui H. (2022). White Matter Change Near Cerebral Microbleeds After mTBI Involves Age and Sex Dependent Cognitive Decline. Innov. Aging..

[43] D’Souza M.M., Kumar M., Choudhary A., Kaur P., Kumar P., Rana P., Trivedi R., Sekhri T., Singh A.K. (2020). Alterations of connectivity patterns in functional brain networks in patients with mild traumatic brain injury: A longitudinal resting-state functional magnetic resonance imaging study. Neuroradiol. J..

[44] Kinnunen K.M., Greenwood R., Powell J.H., Leech R., Hawkins P.C., Bonnelle V., Patel M.C., Counsell S.J., Sharp D.J. (2011). White matter damage and cognitive impairment after traumatic brain injury. Brain..

[45] Niogi S.N., Mukherjee P., Ghajar J., Johnson C.E., Kolster R., Lee H., Suh M., Zimmerman R.D., Manley G.T., McCandliss B.D. (2008). Structural dissociation of attentional control and memory in adults with and without mild traumatic brain injury. Brain..

[46] Li L., Liang J., Fu H. (2021). An update on the association between traumatic brain injury and Alzheimer’s disease: Focus on Tau pathology and synaptic dysfunction. Neurosci. Biobehav. Rev..

[47] Hoffman J., Yu J., Kirstein C., Kindy M.S. (2020). Combined Effects of Repetitive Mild Traumatic Brain Injury and Alcohol Drinking on the Neuroinflammatory Cytokine Response and Cognitive Behavioral Outcomes. Brain Sci..

[48] Chen C.J., Wu C.H., Liao Y.P., Hsu H.L., Tseng Y.C., Liu H.L., Chiu W.T. (2012). Working memory in patients with mild traumatic brain injury: Functional MR imaging analysis. Radiology.

[49] Rostami E., Davidsson J., Ng K.C., Lu J., Gyorgy A., Walker J., Wingo D., Plantman S., Bellander B.M., Agoston D.V. (2012). A Model for Mild Traumatic Brain Injury that Induces Limited Transient Memory Impairment and Increased Levels of Axon Related Serum Biomarkers. Front. Neurol..

[50] Cicerone K.D., Goldin Y., Ganci K., Rosenbaum A., Wethe J.V., Langenbahn D.M., Malec J.F., Bergquist T.F., Kingsley K., Nagele D. (2019). Evidence-Based Cognitive Rehabilitation: Systematic Review of the Literature from 2009 through 2014. Arch Phys Med Rehabil..

[51] Reddy C.C., Collins M., Lovell M., Kontos A.P. (2013). Efficacy of amantadine treatment on symptoms and neurocognitive performance among adolescents following sports-related concussion. J. Head. Trauma. Rehabil..

[52] Clarke G.J.B., Skandsen T., Zetterberg H., Follestad T., Einarsen C.E., Vik A., Mollnes T.E., Pischke S.E., Blennow K., Håberg A.K. (2024). Longitudinal Associations Between Persistent Post-Concussion Symptoms and Blood Biomarkers of Inflammation and CNS-Injury After Mild Traumatic Brain Injury. J. Neurotrauma.

[53] Bazarian J.J., Abar B., Merchant-Borna K., Pham D.L., Rozen E., Mannix R., Kawata K., Chou Y., Stephen S., Gill J.M. (2023). Effects of Physical Exertion on Early Changes in Blood-Based Brain Biomarkers: Implications for the Acute Point of Care Diagnosis of Concussion. J. Neurotrauma..

[54] Sun Z.-L., Feng D.-F. (2013). Biomarkers of cognitive dysfunction in traumatic brain injury. J. Neural Transm..

[55] Vaughn M.N., Winston C.N., Levin N., Rissman R.A., Risbrough V.B. (2022). Developing Biomarkers of Mild Traumatic Brain Injury: Promise and Progress of CNS-Derived Exosomes. Front. Neurol..

[56] Ganesh A., Galetta S. (2021). Editors’ Note: Blood Biomarkers of Traumatic Brain Injury and Cognitive Impairment in Older Veterans. Neurology.

[57] Peltz C.B., Kenney K., Gill J., Diaz-Arrastia R., Gardner R.C., Yaffe K. (2020). Blood biomarkers of traumatic brain injury and cognitive impairment in older veterans. Neurology.

[58] Shen X., Li A., Zhang Y., Dong X., Shan T., Wu Y., Jia J., Hu Y. (2013). The effect of different intensities of treadmill exercise on cognitive function deficit following a severe controlled cortical impact in rats. Int. J. Mol. Sci..

[59] Traeger J., Hoffman B., Misencik J., Hoffer A., Makii J. (2020). Pharmacologic Treatment of Neurobehavioral Sequelae Following Traumatic Brain Injury. Crit. Care Nurs. Q..

[60] Prins M.L., Hovda D.A. (2009). The effects of age and ketogenic diet on local cerebral metabolic rates of glucose after controlled cortical impact injury in rats. J. Neurotrauma..

[61] Markovic S.J., Fitzgerald M., Peiffer J.J., Scott B.R., Rainey-Smith S.R., Sohrabi H.R., Brown B.M. (2021). The impact of exercise, sleep, and diet on neurocognitive recovery from mild traumatic brain injury in older adults: A narrative review. Ageing Res. Rev..

